# NAMSTCD: A Novel Augmented Model for Spinal Cord Segmentation and Tumor Classification Using Deep Nets

**DOI:** 10.3390/diagnostics13081417

**Published:** 2023-04-14

**Authors:** Ricky Mohanty, Sarah Allabun, Sandeep Singh Solanki, Subhendu Kumar Pani, Mohammed S. Alqahtani, Mohamed Abbas, Ben Othman Soufiene

**Affiliations:** 1 School of Information System, ASBM University, Bhubaneswar 754012, Odisha, India; 2 Department of Medical Education, College of Medicine, Princess Nourah bint Abdulrahman University, P.O. Box 84428, Riyadh 11671, Saudi Arabia; 3 Department of Electronics and Communication Engineering, Birla Institute of Technology, Mesra 835215, Jharkhand, India; 4 Krupajal Engineering College, Bhubaneswar, Pipili 752104, Odisha, India; 5 Radiological Sciences Department, College of Applied Medical Sciences, King Khalid University, Abha 61421, Saudi Arabia; 6 BioImaging Unit, Space Research Centre, University of Leicester, Michael Atiyah Building, Leicester LE1 7RH, UK; 7 Electrical Engineering Department, College of Engineering, King Khalid University, Abha 61421, Saudi Arabia; 8 PRINCE Laboratory Research, ISITcom, Hammam Sousse, University of Sousse, Sousse 4000, Tunisia

**Keywords:** spinal, cord, segments, tumor, classification, segmentation, convolutional, neural network, mask regions

## Abstract

Spinal cord segmentation is the process of identifying and delineating the boundaries of the spinal cord in medical images such as magnetic resonance imaging (MRI) or computed tomography (CT) scans. This process is important for many medical applications, including the diagnosis, treatment planning, and monitoring of spinal cord injuries and diseases. The segmentation process involves using image processing techniques to identify the spinal cord in the medical image and differentiate it from other structures, such as the vertebrae, cerebrospinal fluid, and tumors. There are several approaches to spinal cord segmentation, including manual segmentation by a trained expert, semi-automated segmentation using software tools that require some user input, and fully automated segmentation using deep learning algorithms. Researchers have proposed a wide range of system models for segmentation and tumor classification in spinal cord scans, but the majority of these models are designed for a specific segment of the spine. As a result, their performance is limited when applied to the entire lead, limiting their deployment scalability. This paper proposes a novel augmented model for spinal cord segmentation and tumor classification using deep nets to overcome this limitation. The model initially segments all five spinal cord regions and stores them as separate datasets. These datasets are manually tagged with cancer status and stage based on observations from multiple radiologist experts. Multiple Mask Regional Convolutional Neural Networks (MRCNNs) were trained on various datasets for region segmentation. The results of these segmentations were combined using a combination of VGGNet 19, YoLo V2, ResNet 101, and GoogLeNet models. These models were selected via performance validation on each segment. It was observed that VGGNet-19 was capable of classifying the thoracic and cervical regions, while YoLo V2 was able to efficiently classify the lumbar region, ResNet 101 exhibited better accuracy for sacral-region classification, and GoogLeNet was able to classify the coccygeal region with high performance accuracy. Due to use of specialized CNN models for different spinal cord segments, the proposed model was able to achieve a 14.5% better segmentation efficiency, 98.9% tumor classification accuracy, and a 15.6% higher speed performance when averaged over the entire dataset and compared with various state-of-the art models. This performance was observed to be better, due to which it can be used for various clinical deployments. Moreover, this performance was observed to be consistent across multiple tumor types and spinal cord regions, which makes the model highly scalable for a wide variety of spinal cord tumor classification scenarios.

## 1. Introduction

The spinal cord is a long, thin, tubular bundle of nerve fibers that extends from the brain down through the vertebral column. It is a part of the central nervous system and plays a crucial role in relaying information between the brain and the rest of the body. The spinal cord is protected by the bony vertebral column, which provides both support and flexibility. The spinal cord is divided into four regions: the cervical region (neck), thoracic region (upper back), lumbar region (lower back), and sacral region (pelvis). Each of these regions has a specific set of nerves that control different parts of the body. Injury to the spinal cord can cause a range of disabilities, depending on the location and severity of the injury. Some common effects of spinal cord injury include paralysis, loss of sensation, and loss of bowel and bladder control. Treatments for spinal cord injury include medication, physical therapy, and surgery, but there is currently no cure for spinal cord injury [1].

**Figure 1:**
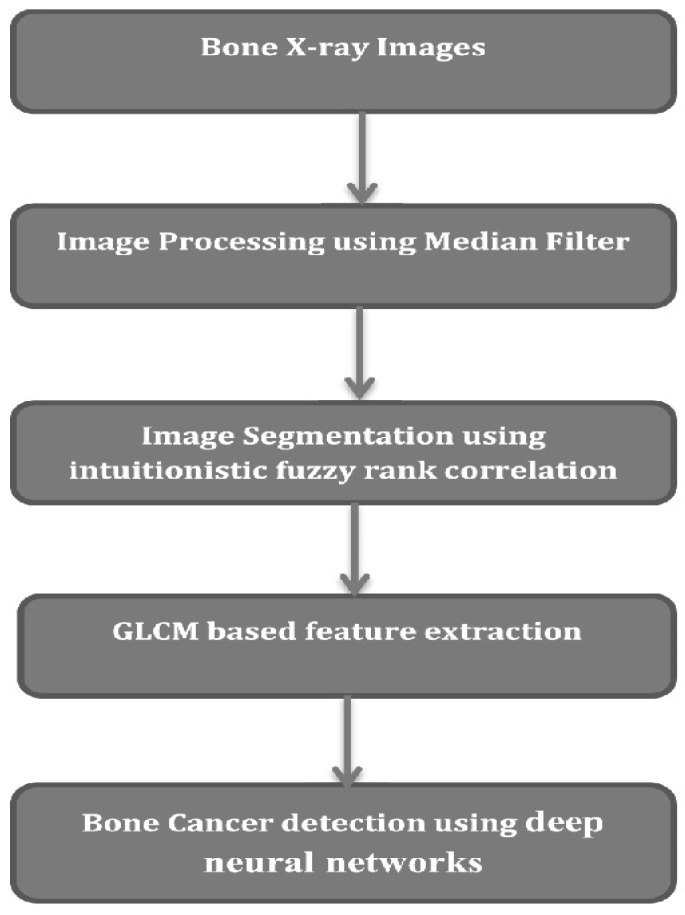
Spinal cord tumor classification model flowchart.

Spinal cord segmentation is a medical image analysis task that involves the automatic or manual delineation of the spinal cord from magnetic resonance imaging (MRI) data. Accurate segmentation of the spinal cord is essential for many clinical applications, such as diagnosis, treatment planning, and monitoring of spinal cord diseases and injuries. Segmentation of the spinal cord can be performed using various techniques, including manual delineation by experts, threshold-based methods, edge detection, region growing, clustering, machine learning, and deep learning-based methods [2]. The choice of method depends on the specific application and the available data. Manual delineation by experts is considered the gold standard for spinal cord segmentation. However, it is time-consuming, tedious, and subject to inter- and intra-rater variability. Automated methods based on image processing and machine learning techniques can provide accurate and reproducible segmentations in a fraction of the time required for manual delineation. Deep learning-based methods, in particular, have shown promising results in spinal cord segmentation, using convolutional neural networks (CNNs) and other deep learning architectures. These methods are data-driven and can learn complex patterns and features from the MRI data, enabling them to generalize well to new data and improve segmentation accuracy. Overall, spinal cord segmentation is a challenging task that requires a combination of expert knowledge, image analysis techniques, and machine learning methods. The accurate segmentation of the spinal cord from MRI data has the potential to improve diagnosis and treatment of spinal cord diseases and injuries. A typical spinal cord image processing model is depicted in Figure 1, wherein different processing components are integrated together for final tumor classification.

Detection of the spinal cord using deep learning algorithms is an active area of research in the field of medical image analysis. Deep learning algorithms are particularly suited to this task, because they can learn complex patterns in large volumes of data, such as medical images, and make accurate predictions. There are different approaches to detecting the spinal cord using deep learning algorithms. One approach is to use convolutional neural networks (CNNs), which are a type of deep learning algorithm that is commonly used for image analysis tasks. CNNs are designed to identify patterns in images by analyzing their local features and spatial relationships. To train a CNN model for spinal cord detection, a large dataset of spinal cord images is needed. This dataset should include images of the spinal cord with different orientations, resolutions, and contrast levels. The images can be obtained from various imaging modalities, such as magnetic resonance imaging (MRI) and computed tomography (CT). Once the dataset is prepared, the CNN can be trained using a supervised learning approach. The CNN learns to classify pixels in the image as either belonging to the spinal cord or not. During training, the CNN adjusts its parameters to minimize the difference between its predicted outputs and the ground truth labels provided in the training dataset. After training, the CNN model can be used to detect the spinal cord in new images. The CNN model takes an image as input and produces a binary mask that highlights the pixels that belong to the spinal cord. The mask can be further processed to extract features of the spinal cord, such as its length, width, and position. Overall, deep learning algorithms such as CNNs have shown promising results for detecting the spinal cord in medical images. However, further research is needed to validate the performance of these algorithms on different datasets and imaging modalities, and to optimize their parameters for clinical use.

It can be observed that segmentation, feature extraction and classification are the major blocks which assist in achieving high-efficiency tumor classification performance [3]. Based on this model flow, a wide variety of spinal cord tumor classification models have been proposed by researchers, and each of them varies in terms of its applicability and performance. In [4], the authors used different ML methods including k-nearest-neighbor, a neural network with radial basis functions, and a naive Bayer classifier to classify vertebral compression fractures as either benign or malignant on T1-weighted sequences. They achieved an AUROC of 0.97 in detecting vertebral fractures and of 0.92 in classifying them as benign or malignant. However, their model was limited by their manual segmentation process (introducing intra- and interobserver variability) and their individual analysis of the vertebral bodies, ignoring relevant information such as the presence of epidural masses. Thomas et al. trained a deep CNN to differentiate between tuberculous and pyogenic spondylitis on axial T2-weighted MRI images, concluding that the algorithm’s performance was comparable to that of three radiologists. They suggested that their model could be used to identify spondylitis as an incidental finding on spine MRI obtained for reasons other than for the assessment of a suspected infection. However, the DL method used in the model needs further validation with a larger-scale study that utilizes multiplanar MR images [5]. The eventual integration of these spinal cord deep learning algorithms into routine clinical practice would open the door to potential improvements in diagnostic sensitivity, treatment monitoring, and patient outcomes, with resultant value added for both clinicians and our patients.

On the basis of the literature described above, this work proposes a new augmented model for spinal cord segmentation and tumor classification using deep nets. The proposed model initially divides each spinal cord image into multiple segments via the MRCNN model and uses these segments to train an ensemble of CNN classifiers. Each of these classifiers is responsible for detecting a particular type of tumor, which assists in improving model scalability and performance. The model initially segments all five spinal cord regions and stores them as separate datasets. These datasets are manually tagged with cancer status and stage based on observations from multiple radiologist experts. Multiple Mask Regional Convolutional Neural Networks (MRCNNs) were trained on various datasets for region segmentation. The results of these segmentations are combined using a combination of VGGNet 19, YoLo V2, ResNet 101, and GoogLeNet models. These models were selected via performance validation on each segment. It was observed that VGGNet-19 was capable of classifying Thoracic and Cervical regions, while YoLo V2 efficiently classified Lumbar regions, ResNet 101 showcased better accuracy for Sacral region classification, and Goog-LeNet classified Coccygeal regions with high accuracy performance. This performance is evaluated in terms of Peak Signal-to-Noise Ratio (PSNR), accuracy, and delay measures in Section 4, and compared with various state-of-the-art models. Based on this comparison, it can be observed that the proposed model is highly scalable for multiple spinal cord regions and showcases better performance w.r.t. existing classification models. Finally, this text concludes with some interesting observations about the proposed model and recommends various methods to further improve its performance.

## 2. Related Work

A wide variety of models have been proposed for spinal cord processing and classification, and every one of them has explicit execution. For example, in the work in [5], limit-based division and Convolutional Neural Network (CNN)-based division models are portrayed. It can be seen that the edge-based model has limited precision, and must be physically tuned for each dataset, while the CNN model is autotuned, and has high division effectiveness, and in this manner can be applied to a wide assortment of datasets. An examination of such models was discussed in [6], from where it can be seen that AI techniques beat direct division models, and consequently are profoundly liked for clinical applications. This model was additionally examined in [7], wherein division repeatability of thoracic spinal muscle morphology was performed by means of deep learning-based characterization strategies. A plan of a comparable model was additionally portrayed in [8], wherein the Statistical Parametric Planning (SPP) structure was depicted. This strategy has great precision and can be utilized for quite a long time with negligible preparation exertions on the client side. In any case, these models require huge delays for preparation, which can be streamlined through utilization of equal handling, or pipelining methods. An illustration of such a high-speed performance model is proposed in [9,10,11], in which scientists utilized a deep learning network with more learning. The fusing of move learning diminishes cold-start issues, and hence, in general, works on Accuracy, Recall, and FMeasure execution. Motivated by this perception, the proposed model additionally utilizes move learning to achieve the profoundly effective division of spinal rope symbolism. Comparatively high-productivity models are proposed in [12,13,14], wherein the analysts utilized scientific change-based completely computerized convolutions, vertebrae division, and Particle Swarm Optimization (PSO) models to achieve high exactness in low postpone division and order tasks. The PSO model will, in general, beat other models because of its minuscule division execution, and capacity to perform order and relapse with high productivity.

The effectiveness of the assessed models should additionally be assessed on bigger datasets. Such examination was discussed in [15,16,17], wherein vertebral estimation, Deep Neural Network (DNN) for injury grouping, and Dense Dilated Convolutions (DDC) are utilized for division and arrangement activities. By displaying different datasets and using the Finite Element Method (FEM) for the division and inspection of spinal lines, the models in [18,19] further assist in developing the arrangement and executing the division. Table 1 shows the main Convolutional Neural Network applications for Deep segmentation models.

## 3. Proposed Model

Based on the literature review, it can be observed that a wide variety of machine learning models have been proposed by researchers for high-efficiency and low-computational-time spinal cord segmentation and tumor classification scenarios. However, these models were developed for particular portions of the spine, making them applicable only in a specific spinal cord segmentation application scenario. To overcome this limitation, a novel augmented model for spinal cord segmentation and tumor classification using deep nets is discussed in this section, wherein segmentation results from Multiple Mask Regional Convolutional Neural Networks (MRCNNs) are combined with VGGNet 19, YoLo V2, ResNet 101, and GoogLeNet classification models.

Multiple Mask Regional Convolutional Neural Networks (MMRCNN) are a type of deep learning architecture used for image recognition and object detection tasks. It is an extension of the popular Faster R-CNN algorithm, which uses a Region Proposal Network (RPN) to generate region proposals for objects in an image, and a Region of Interest (ROI) pooling layer to extract features from the proposed regions. MMRCNN improves upon Faster R-CNN by introducing multiple masks for each region proposal, instead of a single ROI pooling layer. These masks are used to refine object boundaries and improve the accuracy of object detection. The network also includes an additional branch for predicting object masks, which helps in segmentation tasks. In MMRCNN, the RPN generates region proposals, which are then passed through several convolutional layers to generate features for each proposed region. These features are then fed into multiple ROI pooling layers, each of which generates a mask for the proposed region. The resulting masks are combined to refine the object boundaries, and the final output is a set of object proposals along with their corresponding masks.

**Figure 2:**
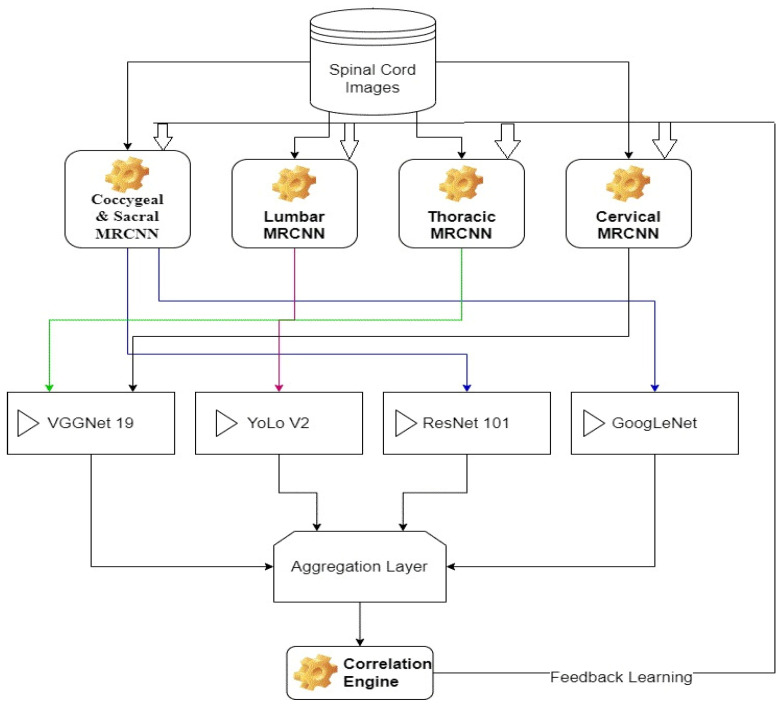
Overall flow of the proposed model.

Each of these models is trained for a particular segment of spinal cord, which assists in achieving better classification performance. The overall flow of the proposed method is depicted in Figure 2, wherein different MRCNNs segmentation models are combined with CNN models to achieve final segmentation. From the flow, it can be observed that a tagged database of spinal cord images is segmented via different MRCNN models, which assists in identification of different regions with better segmentation performance. These segmented images are classified using an augmentation of VGGNet 19, YoLo V2, ResNet 101, and GoogLeNet classifiers. Data augmentation is a technique used in deep learning to increase the size of a dataset by generating new samples from existing ones, typically through a series of random transformations. This technique helps to improve the robustness and generalization ability of deep learning models by introducing more variations and diversity into the training data. The results of these classifiers are combined using an aggregation layer, which assists in the final estimation of spinal cord conditions. These conditions are validated via a correlation layer, which is used for continuous database update operations. Due to use of this continuous update layer, the model is capable of incrementally improving its performance with respect to the number of evaluations.

Each of these blocks, along with their internal design details are discussed in separate sub-sections of this text. Researchers can implement these models in part(s) or as a full system depending upon their application requirements.

### 3.1. Design of the MRCNN Model for Segmentation of Different Spinal Cord Regions

The input spinal cord images are initially segmented using a MRCNN model that uses eXplanation with Ranked Area Integrals (XRAI) for region-based analysis. Ranked area integrals are a type of mathematical technique used to calculate the area under a curve. The basic idea behind this technique is to divide the region under the curve into small strips or rectangles, calculate the area of each strip, and then add up the areas of all the strips to obtain the total area.

Using the XRAI method, medical images are segmented, and their Regions of Interest (RoIs) are extracted from raw images. To perform this task, the entropy for each pixel is evaluated via convolutional processing and bit plane slicing models. To extract these features, convolutional operations are performed, which assist in high-variance feature representation.

**Figure 3:**
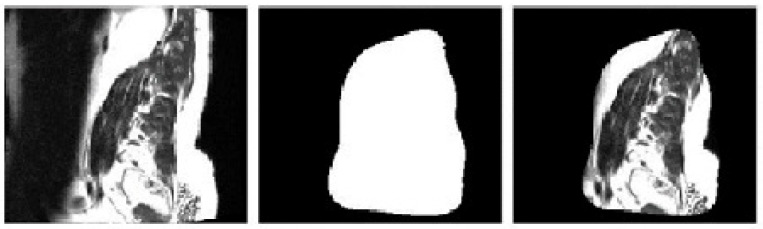
Input spinal cord image, its Saliency Mask, and final Saliency Map image.

A high-variance feature representation refers to a set of features in a dataset that exhibit a wide range of values or variability. In other words, the values of these features can vary significantly from one data point to another. High-variance features can be useful in some machine learning tasks, such as classification or regression, because they may contain important information for distinguishing between different classes or predicting an outcome. However, they can also pose challenges, as they may be more susceptible to overfitting, where a model learns to fit the noise in the training data rather than the underlying patterns. In this paper, we used this technique to reduce redundancies and unique values in image features so high variance feature representation is used. Image pixels are initially processed via an entropy evaluation layer, which estimates energy levels of pixels. Entropy of images is evaluated via Equation (1), wherein pixel levels and their logarithmic intensities are averaged to form final image entropy.

(1) $$E_{f_{i}}=-\sum_{r=1}^{N}\sum_{c=1}^{M}\frac{N*M}{p\left({I_{r,c}}_{i}\right)*\log\left(\frac{1}{p\left({I_{r,c}}_{i}\right)}\right)}$$

where $p\left({I_{r,c}}_{i}\right)$ represents image pixel value at r,c location, and i represents slice number of the raw input image. Based on these entropy levels, pixels with values above $E_{f_{i}}$ are termed foreground, while others are marked as background and supressed from the output. This process is performed at slice level, and these slices are combined to form the final segmented image set. This process is termed Saliency extraction, and an example of it can be observed in Figure 3, wherein the input image and its corresponding saliency map are visualized.

The extracted regions are processed via a Masked Region CNN model, which assists in classification of each pixel set into different spinal cord segments. Masked Region CNN (Convolutional Neural Network) is a type of neural network that is designed to process images or visual data with a particular focus on regions of interest (ROIs) in the image. It is also known as Mask R-CNN. Mask R-CNN is an extension of Faster R-CNN, which is a two-stage object detection algorithm that uses a region proposal network (RPN) to generate candidate regions in an image, followed by a classification and regression network to classify each region and refine the bounding box coordinates. Mask R-CNN builds on top of this architecture by adding a third branch to the network that generates a binary mask for each ROI, indicating which pixels belong to the object and which do not. In addition to object detection and instance segmentation, Mask R-CNN can also be used for semantic segmentation by treating each object in the image as a separate class. This allows the network to assign a semantic label to each pixel in the image, which can be useful for tasks such as image segmentation and scene understanding. Mask R-CNN has achieved state-of-the-art results on several benchmark datasets for object detection and instance segmentation, and it has been widely adopted in computer vision research and applications.

**Figure 4:**
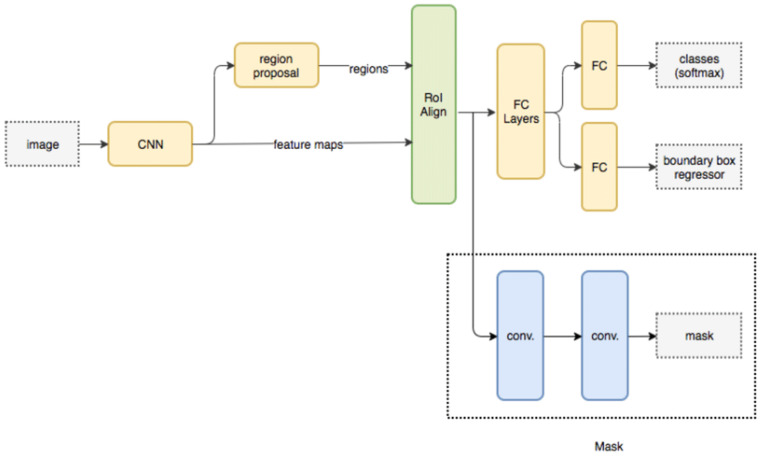
Masked Region CNN model for pixel-level classification for segment extraction.

The masked RCNN model is depicted in Figure 4, wherein different convolutional layers, along with their interconnections, are visualized. It can be observed that the MRCNN Model is trained for different types of spinal cord segments, and then evaluated at pixel level. Due to which individual Region Proposal Networks (RPNs) are trained and evaluated for different spinal cord segments. Each RPN layer consists of different inception and mapping modules, which assist in separating pixels of one segment from others.

**Figure 5:**
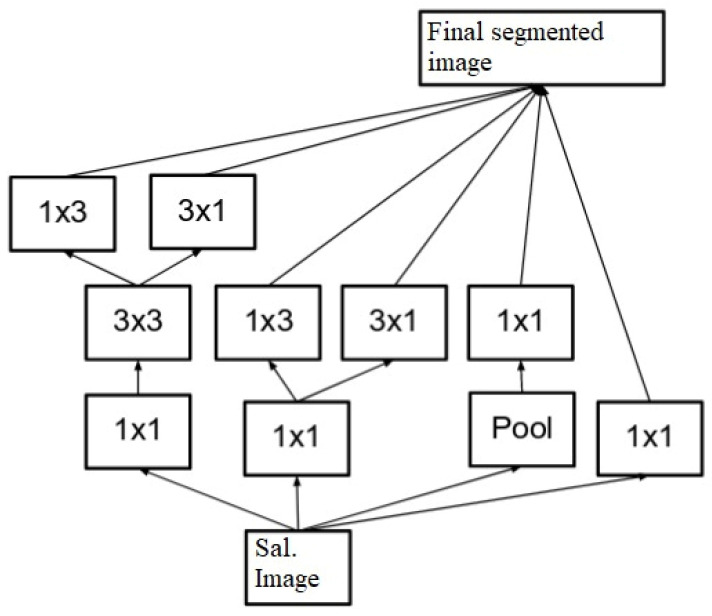
Design of the Inception Model.

To perform this task, masks for different regions are convoluted with the Saliency Map image, which assists in obtaining segment-level images. This assists in separating the input image into different sub-components, which increases the efficiency of the tumor classification process. The results of RPN are evaluated using Equation (2):

(2) $$Seg_{out}\left(p\right)=\sum \log\left(SM\left(I\right)*Mask\left(p\right)\right)$$

where $SM\left(I\right),\;and\;Mask(p)$ represent the Saliency Map image and the Mask for the current part of spine segment, respectively. The equation signifies the summation of the Saliency Map image and the Mask for the current part of spine segment. Due to the complexity of spinal structures, multiple masks are combined to form the final segmented ($Seg_{out}$) image. These masks, along with their filter-level concatenation process, can be visualized using Figure 5, where masks of different shapes and sizes are combined to form the final output image set.

The final filter mask can be represented via Equation (3), wherein different smaller sized masks are combined for each region, which assists in achieving better segmentation performance.

(3) $$Mask\left(p\right)=\sqrt{\frac{\left(a*B\left(p\right)+c\right)*\left(\frac{P\left(p\right)}{f}+d\right)}{f}}$$

where a,c,d, and f represent mask-level constants, and can be tuned as per the input dataset, $P\left(p\right)\;and\;B(p)$ represent the pixel-level mask and the binary mask for the current part of the spinal cord. These pixel-level masks are pre-set by the MRCNN model and are used for the final segmentation process. Multiple RCNN modules are connected individually, assisting in the extraction of different spinal cord segments. All of these masks and their generated segments are individually given to different CNN models for classification of segment level tumors.

### 3.2. Design of the CNN Model for Tumor Classification

**Figure 6:**
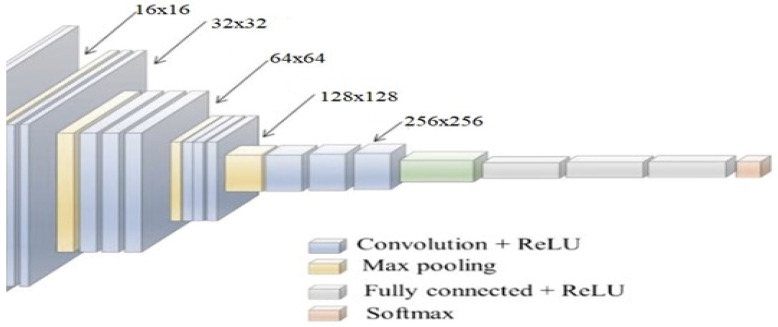
A typical CNN model used for classification of spinal cord regions.

Individual extracted spinal cord segments are given to different CNN models for tumor classification. During evaluation, it was observed that VGGNet-19 showcased higher efficiency for the classification of thoracic tumors and cervical-region tumors, YoLo V2 had better performance for lumbar-region tumors, ResNet 101 achieved higher accuracy for sacral-region tumors, and GoogLeNet performed better for coccygeal-region tumors, achieving high performance accuracy. A typical CNN model is depicted in Figure 6, wherein different convolutional operations are cascaded with maximum feature pooling (Max Pool) and drop-out layers. A Max Pool layer is a type of pooling layer commonly used in convolutional neural networks (CNNs) for image recognition tasks.

The main function of a max pooling layer is to reduce the spatial dimensionality (i.e., the height and width) of the input volume (i.e., the output of a convolutional layer) while retaining the most important features. It works by sliding a fixed-size window (called the pooling window or filter) over the input volume and outputting the maximum value within each window. In this paper, an elaboration of the design of the max pooling layer is given in order to obtain a clear picture of the convolutional neural network. It works by sliding a fixed-size window (called the pooling window or filter) over the input volume and outputting the maximum value within each window.

To process spinal cord segments, the CNN models extract a large number of convolutional features, which assist in obtaining a high-accuracy image representation of the input images. Based on these convolutions, different statistical measures including mean, max, standard deviation, kurtosis, entropy, variance, and correlation coefficient values are estimated at block level. Thus, each input image segment is divided into different blocks, and each block is processed by means of windowing and padding constants. An instance of these convolutions is evaluated in Equation (4), wherein input image pixels are activated via Leaky ReLU (rectilinear unit) kernels.

(4) $$Conv_{out_{i,j}}=\sum_{a=-\frac{m}{2}}^{\frac{m}{2}}\sum_{b=-\frac{n}{2}}^{\frac{n}{2}}LReLU\left(\frac{m}{2}+a,\frac{n}{2}+b\right)*SC_{comp}\left(i-a,j-b\right)$$

where $LReLU,SC_{comp}$ represents the Leaky ReLU kernel and the spinal cord component, respectively, while m,n represent the window size across the rows and columns of the input image, respectively. These convolutional features are evaluated for each window size and assist in the estimation of a large number of features. The features extracted from each convolutional layer of the CNN are checked to reveal some internal working mechanisms of the CNN and explain the specific meanings of some features. Due to the variation in different padding, stride, and kernel sizes, this model is able to extract a large number of features from any spinal cord image. However, with increasing numbers of convolutional layers, the total number of features extracted per spinal cord segment also increases.

The number of features extracted by these layers is evaluated using Equation (5):

(5) $$N_{f_{extract}}=\frac{N_{f_{input}}+2*p-k}{s}+1$$

where $N_{f_{extract}}\;and\;N_{f_{input}}$ represent the extracted and input features for the given convolutional layer, p,k,s represent the padding size used during convolution, the stride size used during convolution, and the kernel size used during convolution in the current layer, respectively.

The features extracted from each convolutional layer of the CNN are checked to reveal some internal working mechanisms of the CNN and explain the specific meanings of some features. Due to the variations in different padding, stride, and kernel sizes, this model is able to extract a large number of features from any spinal cord image. However, with increasing numbers of convolutional layers, the total number of features extracted per spinal cord segment also increases. To cut these redundant positions, each convolutional layer is followed by a Max Pool layer. These layers calculate the variance threshold for each extracted feature set and choose the features with the highest variation levels on the basis of this variance threshold. The variance threshold for each Max Pool layer is evaluated using Equation (6):

(6) $$f_{th}={\left(\sum_{x\in X_{k}}\frac{s_{i}}{x^{p_{v}}}\right)}^{p_{v}^{-1}}$$

where $s_{i}$ represents variance for the given feature set, x represents extracted features, and $p_{v}$ represents the probability of variance for the given feature set.

This probability is tuned during each iteration and assists in achieving better feature extraction performance. This performance is enhanced through the use of different-sized convolution layers. In this case, layers with sizes of 3 × 3 × 512, 7 × 7 × 256, 16 × 16 × 128, and 32 × 32 × 64 and 64 × 64 × 32 are used by the CNN models to extract a large number of highly variant features. These features are processed via a Fully Connected Neural Network (FCNN), which assists in the identification of tumor classes. The model is able to differentiate between tumor and non-tumor classes, due to which it is used in binary classification mode, which assists in achieving higher classification performance when compared with sparse categorical classifications. To obtain the final class, Equation (7) is used, wherein a Softmax-based activation layer is deployed to improve feature segregation into tumor and non-tumor classes. Softmax is a mathematical function that takes a vector of real numbers as input and returns a probability distribution over the elements of that vector. It is commonly used in machine learning and deep learning to convert a set of scores or logits into probabilities that can be used for classification tasks.

(7) $$T_{out}=SoftMax\left(\sum_{i=1}^{N_{f}}f_{i}*w_{i}+b\right)$$

where $f_{i}$ represents the values of the extracted convolutional feature vectors, $w_{i}$ represents weight, b represents bias, and $N_{f}$ represents the total features extracted by the convolutional layers. Each of these classes is evaluated for the coccygeal, sacral, lumbar, thoracic, and cervical regions individually. These classes are given to an aggregation layer, which assists in the identification of the final tumor state for the spinal cord. The design of this layer is discussed in the next section of this text.

### 3.3. Design of Aggregation Layer with Correlation Engine for Continuous Performance Enhancement

The CNN layer assists in the identification of cancer status for different regions of the spinal cord. These status values are aggregated using Equation (8) to obtain final cancer spreading probability.

(8) $$P\left(C_{spread}\right)=\frac{\left\{\begin{array}{c} T_{out}\left(Coccygeal\right)*W_{Coccygeal}+\\ T_{out}\left(Sacral\right)*W_{Sacral}+\\ T_{out}\left(Lumbar\right)*W_{Lumbar}+\\ T_{out}\left(Thoracic\right)*W_{Thoracic}+\\ T_{out}\left(Cervical\right)*W_{Cervical} \end{array}\right\}}{5}$$

where $W_{i}$ represents the weight of the current spinal cord segment, and is estimated using Equation (9):

(9) $$W_{i}=\frac{L_{i}}{\sum_{j=1}^{5}L_{j}}$$

where $L_{i}$ represents approximate length of spinal cord region for the given patient, and is estimated using Equation (10):

(10) $$L_{i}=\frac{N_{p_{i}}}{\sum_{l=1}^{5}N_{p_{l}}}$$

where $N_{p_{l}}$ represents the count of the total number of pixels for a given spinal cord segment. On the basis of this evaluation, the probability of cancer spread across the entire spinal cord is evaluated. These results are correlated with the actual spread probability values (C) using Equation (11):

(11) $$C=\frac{\sum_{i=1}^{N}P_{actual_{i}}-P_{obtained_{i}}}{\sqrt{\sum_{i=1}^{N}{\left(P_{actual_{i}}-P_{obtained_{i}}\right)}^{2}}}$$

where $P_{actual},\;and\;P_{obtained}$ represent the actual and obtained values of probability, while N represents the total number of images used to perform this evaluation. If correlation with the actual spread probability value is greater than 0.999, then this image of the spinal cord regions is added to the training set, on the basis of which the model is able to continuously improve its performance in terms of both accuracy and precision. Estimation of this performance is discussed in the next section of this paper, wherein the performance of the proposed model is compared with various state-of-the-art approaches.

## 4. Results and Comparison

In order to estimate the classification performance of the proposed NAMSTCD model, spinal cord images from multiple Mendeley datasets and their ground truth images were used. These data were obtained from https://data.mendeley.com/datasets/zbf6b4pttk/2 (accessed on 25 December 2022) [20] and are freely available under an open-source license. The dataset was evaluated using the proposed NAMSTCD model, and the values for segmentation peak signal-to-noise ratio (PSNR), classification accuracy, and computational delay were evaluated and compared with the values obtained using CNN [5], SPM [8], and DNN [16]. The classification accuracy was evaluated using Equation (12), as follows:

(12) $$A=\frac{N_{correct}}{N_{total}}*100$$

where *N_correct* and *N_total* represent the total number of correctly classified images and the total number of rated images, respectively. The entire dataset of 5000 images was split 60:10:30 for training, validation and testing, respectively. The accuracy is listed in Table 2, below.

**Figure 7:**
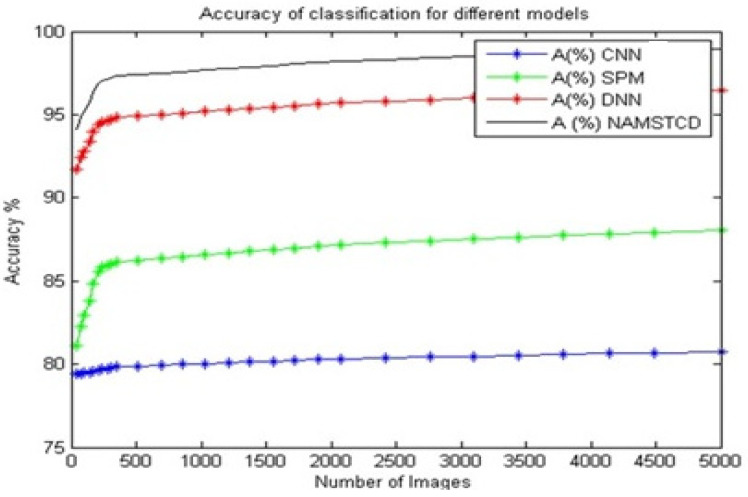
Accuracy of classification for different models.

From Table 3 and Figure 7, it can be observed that the proposed model has an accuracy that is 18.1% better than that of CNN [5], 10.5% better than that of SPM [8], and 2.3% better than that of DNN [16] on the same dataset.

This suggests that the proposed model is highly efficient for large-scale deployments and can be used in real-time clinical classification applications. Similarly, the PSNR during segmentation was evaluated for CNN [5], SPM [8] and DNN [16], and compared with the values obtained for the proposed model; these values are shown in Table 3, below.

**Figure 8:**
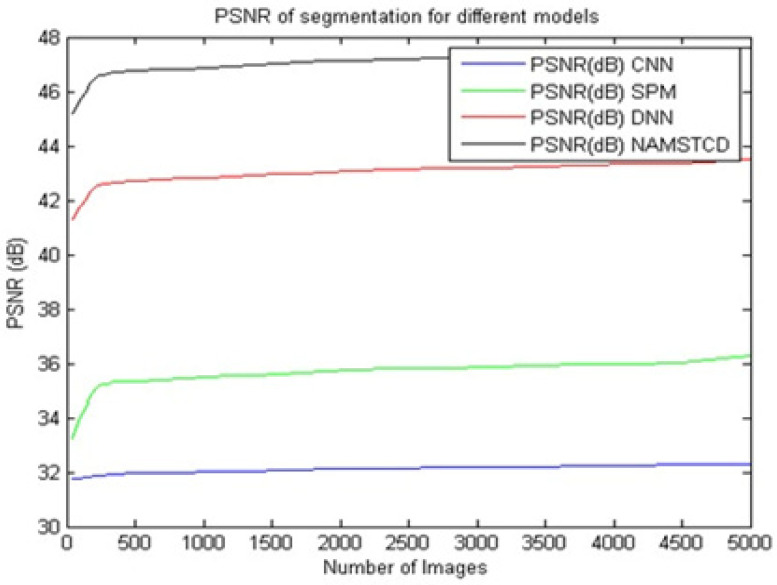
PSNR of classification for different models.

It can be seen from Table 3 and Figure 8 that the proposed model presents an improvement in PSNR of 14.6 dB compared to CNN [5], an improvement of 10.5 dB over SPM [8] and an improvement of 3.4 dB over DNN [16] on the same dataset. This improvement in PSNR is due to the combination of XRAI and MRCNN, which helps to perform fine-tuned segmentation. This suggests that the proposed model is highly efficient for large-scale deployment and can be used to perform real-time clinical segmentation.

Similarly, the computational delay during classification was evaluated for CNN [5], SPM [8] and DNN [16], and compared with the proposed model. Using MATLAB 2019 b, the computational delay was taken into account. These values are shown in Table 4, below.

**Figure 9:**
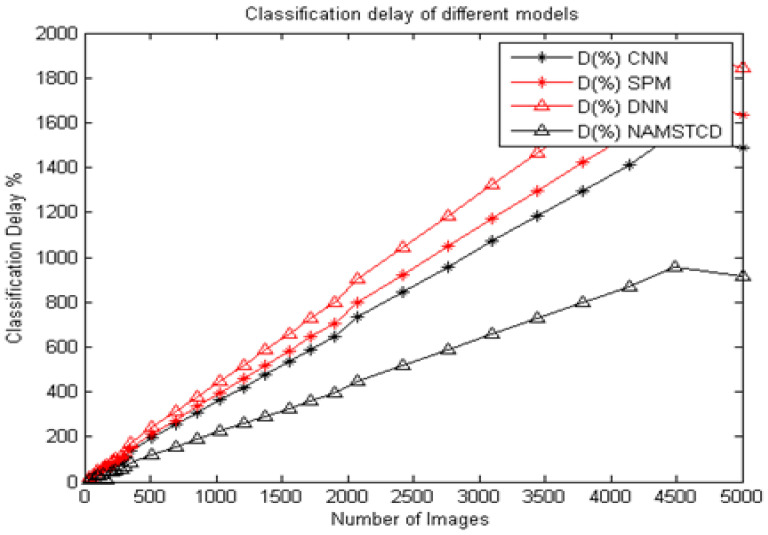
Classification delay of different models.

It can be seen from Table 4 and Figure 9 that the proposed model has a computational delay that is 25.1% lower than CNN [5], 31.4% lower delay than SPM [8] and 39.3% lower than DNN [16] on the same data set. This reduction in computational delay is due to the combination of XRAI and MRCNN, which helps to achieve fine-tuned segmentation, thereby reducing the overall computational delay arising from classification and post-processing operations.

These improvements make the proposed model useful for a wide range of real-time clinical classification applications. It also identifies the likelihood of tumor spread, which further helps to improve its scalability and applicability to a wide range of clinical scenarios.

## 5. Conclusions

The proposed NAMSTCD model uses MRCNN-based segmentation in combination with CNN classification to assist in region-based image extraction and cancer probability analysis. The model also uses a continuous learning mechanism that helps to gradually improve the classification performance. Due to these characteristics, the proposed model is able to achieve a classification accuracy of 98.95%, a segmentation PSNR of 47.62 dB, and a delay of less than 900 s for input sets with a large number of images. This performance was compared with various state-of-the-art models, and it was observed that the proposed model had an accuracy that was 18.1% better than CNN [5], 10.5% better than SPM [8] and 2.3% better than DNN [16] on the same dataset. It also demonstrated an improvement in PSNR of 14.6 dB compared to CNN [5], an improvement of 10.5 dB compared to SPM [8] and an improvement of 3.4 dB over DNN [16], with a delay 25.1% lower than CNN [5], 31.4% lower than SPM [8] and 39.3% lower than DNN [16] on the same dataset. This improvement was achieved through the development of better segmentation, classification and post-processing model designs. In the future, researchers can verify the performance of this model on other datasets, which will help to estimate its scalability and applicability to a wider range of images. In addition, researchers can also replace CNN models with recurrent NN (RNN) models to further improve classification the capabilities for larger datasets. This will help achieve better deployment performance for different clinical scenarios.

## Figures and Tables

**Table 1:** Summary of literature.

Reference	Technique	Dataset	Accuracy (%)
[5]	CNN	Mendeley	80.72
[6]	Mask RCNN	CSI 2014	93.05
[7]	U-Net	MRI scans	96.23
[8]	SPM	Mendeley	88.03
[9]	SegNet	VerSe2020	93.37
[10]	Iterative FCN	X-ray images	96.66
[12]	DABU-Net DenseMCW1-Net	MRI scans	95.45
[13]	CNN	MRI scans	88.23
[14]	Mask RCNN	CSI 2016	96.77
[15]	CNN	CSI 2016	89.36
[16]	DNN	Mendeley	96.43
[17]	FCN deep probabilistic regression	CSI 2016	95.58
[18]	CNN	CSI 2016	90.48
[19]	CNN	CSI 2016	92.47

**Table 2:** Percentage accuracy of image classification using different models.

Number of Images	Accuracy (%)			
	CNN [5]	SPM [8]	DNN [16]	NAMSTCD
35	79.39	81.11	91.72	94.11
70	79.44	82.26	92.40	94.82
103	79.47	82.95	92.81	95.23
138	79.52	83.84	93.35	95.78
172	79.58	84.84	93.95	96.41
207	79.64	85.58	94.41	96.88
241	79.69	85.84	94.59	97.06
276	79.73	85.92	94.66	97.13
310	79.78	86.00	94.73	97.21
345	79.83	86.13	94.84	97.31
517	79.88	86.25	94.93	97.41
690	79.93	86.35	95.01	97.49
862	79.98	86.45	95.10	97.58
1034	80.03	86.56	95.19	97.68
1207	80.08	86.66	95.28	97.77
1379	80.13	86.77	95.37	97.86
1552	80.18	86.87	95.45	97.95
1724	80.23	86.98	95.54	98.04
1897	80.28	87.08	95.63	98.13
2069	80.33	87.19	95.72	98.22
2414	80.37	87.29	95.81	98.31
2759	80.43	87.40	95.89	98.41
3103	80.48	87.51	95.98	98.49
3448	80.53	87.61	96.07	98.58
3793	80.57	87.72	96.16	98.67
4138	80.63	87.82	96.25	98.76
4483	80.67	87.93	96.34	98.86
5000	80.72	88.03	96.43	98.95

**Table 3:** PSNR of segmentation using different models.

Number of Images	PSNR (dB)			
	CNN [5]	SPM [8]	DNN [16]	NAM STCD
35	31.76	33.25	41.27	45.17
70	31.77	33.73	41.58	45.51
103	31.78	34.01	41.76	45.71
138	31.81	34.37	42.01	45.97
172	31.83	34.78	42.28	46.27
207	31.86	35.09	42.48	46.50
241	31.88	35.20	42.56	46.59
276	31.89	35.23	42.59	46.63
310	31.92	35.26	42.63	46.66
345	31.94	35.32	42.68	46.71
517	31.95	35.36	42.72	46.76
690	31.97	35.40	42.76	46.80
862	31.99	35.44	42.79	46.84
1034	32.01	35.48	42.83	46.88
1207	32.03	35.53	42.87	46.93
1379	32.05	35.57	42.92	46.97
1552	32.07	35.62	42.96	47.02
1724	32.09	35.66	42.99	47.06
1897	32.11	35.71	43.03	47.11
2069	32.13	35.75	43.07	47.15
2414	32.15	35.79	43.12	47.19
2759	32.17	35.83	43.16	47.23
3103	32.19	35.87	43.19	47.27
3448	32.21	35.92	43.23	47.32
3793	32.23	35.96	43.27	47.37
4138	32.25	36.00	43.32	47.41
4483	32.27	36.05	43.35	47.45
5000	32.29	36.27	43.50	47.62

**Table 4:** Computational delay of different models.

Number of Images	Computational Delay (s)			
	CNN [5]	SPM [8]	DNN [16]	NAM STCD
35	16.84	17.37	20.00	10.00
70	27.89	28.95	33.16	16.75
103	38.95	41.05	46.84	23.50
138	50.00	53.16	60.53	30.50
172	61.58	65.79	74.74	37.50
207	72.63	78.42	88.95	44.25
241	83.68	90.53	102.63	51.00
276	94.74	103.16	116.32	58.00
310	105.79	115.26	130.53	65.00
345	139.47	151.58	172.11	85.50
517	195.79	212.63	241.05	119.75
690	252.11	273.68	310.00	154.25
862	307.89	335.26	378.95	188.75
1034	364.21	396.84	448.42	223.25
1207	420.53	458.42	517.89	257.75
1379	476.84	520.00	587.37	292.25
1552	533.16	581.58	657.37	327.00
1724	589.47	643.68	727.37	361.75
1897	646.32	705.79	797.37	396.50
2069	731.05	798.95	902.11	448.75
2414	843.68	923.16	1041.58	518.25
2759	956.84	1047.37	1181.58	588.00
3103	1070.53	1172.11	1322.11	657.75
3448	1183.68	1297.37	1462.63	727.50
3793	1296.84	1422.63	1603.16	797.75
4138	1410.53	1547.89	1744.21	868.00
4483	1552.63	1704.74	1920.53	955.50
5000	1489.79	1634.74	1842.18	916.44

## Data Availability

The datasets used during the current study are available from the corresponding author on reasonable request.
